# Production of Multiple Brain-Like Ganglioside Species Is Dispensable for Fas-Induced Apoptosis of Lymphoid Cells

**DOI:** 10.1371/journal.pone.0019974

**Published:** 2011-05-24

**Authors:** Iuliana Popa, Nicole Therville, Stéphane Carpentier, Thierry Levade, Olivier Cuvillier, Jacques Portoukalian

**Affiliations:** 1 Laboratoire de Recherche Dermatologique, EA4169 Université de Lyon-1, Hôpital Edouard Herriot, Lyon, France; 2 Institut National de la Santé et de la Recherche Médicale (INSERM) UMR1037, Toulouse, France; 3 Centre de Recherches en Cancérologie de Toulouse, Université de Toulouse, Toulouse, France; 4 Laboratoire de Biochimie Métabolique, Institut Fédératif de Biologie, CHU Purpan, Toulouse, France; 5 Centre National de la Recherche Scientifique (CNRS), Institut de Pharmacologie et de Biologie Structurale (IPBS), Toulouse, France; 6 Université de Toulouse, Toulouse, France; University of Hong Kong, Hong Kong

## Abstract

Activation of an acid sphingomyelinase (aSMase) leading to a biosynthesis of GD3 disialoganglioside has been associated with Fas-induced apoptosis of lymphoid cells. The present study was undertaken to clarify the role of this enzyme in the generation of gangliosides during apoptosis triggered by Fas ligation. The issue was addressed by using aSMase-deficient and aSMase-corrected cell lines derived from Niemann-Pick disease (NPD) patients. Fas cross-linking elicited a rapid production of large amounts of complex a- and b-series species of gangliosides with a pattern and a chromatographic behavior as single bands reminiscent of brain gangliosides. The gangliosides were synthesized within the first ten minutes and completely disappeared within thirty minutes after stimulation. Noteworthy is the observation that GD3 was not the only ganglioside produced. The production of gangliosides and the onset of apoptotic hallmarks occurred similarly in both aSMase-deficient and aSMase-corrected NPD lymphoid cells, indicating that aSMase activation is not accountable for ganglioside generation. Hampering ganglioside production by inhibiting the key enzyme glucosylceramide synthase did not abrogate the apoptotic process. In addition, GM3 synthase-deficient lymphoid cells underwent Fas-induced apoptosis, suggesting that gangliosides are unlikely to play an indispensable role in transducing Fas-induced apoptosis of lymphoid cells.

## Introduction

The cross-linking of the surface receptor Fas (CD95) triggers apoptosis in a variety of cell types, which is associated with an increase of intracellular ceramide levels [1]–[5]. On the other hand, it has been shown that the synthesis and transient accumulation of GD3 ganglioside is required for the progression of apoptotic signals induced by anti-Fas antibodies [6], [7] and membrane-permeable ceramides [6], [8] in lymphoid cells. GD3 results from the addition of a second sialic acid to the one present on GM3, mediated by the action of GD3 synthase (alpha-2,8-sialyltransferase), a transmembrane type II protein of about 40 kDa resident in the early Golgi. A working model has been proposed in which ceramide synthesis mediated by an acid sphingomyelinase (aSMase) would induce the accumulation of the disialoganglioside GD3 [9]. Indeed, Testi and co-workers claimed that Epstein-Barr virus (EBV)-transformed lymphoblasts from Niemann-Pick disease (NPD) patients, which have an inherited deficiency in aSMase activity, displayed an impaired Fas-induced apoptosis and failed to produce GD3, whereas aSMase-reconstituted NPD lymphoblasts could accumulate GD3 and undergo efficient Fas-mediated apoptosis [9]. Neosynthesized GD3 ganglioside is then thought to mediate apoptosis by localizing in mitochondria [6], [8]. These phenomena could be prevented by blocking GD3 synthase expression, indicating that its *de novo* synthesis is necessary [6]. Such a model has been mostly validated by data derived from the addition of high concentrations of GD3 (in the form of micelles) to cell culture suspensions incorporating subcellular organelles (e.g., mitochondria) with a wrong topology [10], or by *in vitro* experiments on purified mitochondria.

Notwithstanding, conflicting reports have been published as for the implication of aSMase in transducing death signals (see reviews [11]–[13]) and in particular in the signaling of Fas-induced apoptosis. Testi and co-workers have shown that NPD lymphoblasts were resistant to Fas-induced apoptosis, and that recovery of wild-type activity in cells (by addition of wild-type aSMase protein to the incubation medium) restored the response to anti-Fas antibodies [9]. In contrast, others have found no difference between NPD lymphoblasts and the corrected ones (by retrovirus-mediated restoration of wild-type aSMase gene), all being fully sensitive to anti-Fas antibodies [14], [15]. Furthermore, Green and co-workers reported that primary cultures of lymphocytes derived from aSMase-deficient (*Smpd1−/−*) mice display no resistance to anti-Fas antibody- or soluble Fas ligand-induced apoptosis [16].

These conflicting results have raised several issues that we proposed to address in this study: (i) is indeed aSMase required to produce GD3 during Fas-induced cell death? (ii) is GD3 the only ganglioside produced in response to apoptotic stresses? and (iii) is ganglioside synthesis either instrumental or a mere epi-phenomenon in the apoptotic process?

## Materials and Methods

### Reagents

Anti-Fas (clone CH-11) antibody was from Beckman-Coulter (Roissy CDG, France). Murine FasL was produced in the supernatant of Neuro-2A cells stably transfected with a plasmid encoding FasL [17]. Recombinant human FasL was a kind gift from Dr. P. Legembre (Rennes, France). C_6_-ceramide and D,L-*threo*-1-phenyl-2-decanoylamino-morpholino-1-propanol (PDMP) were from Biomol (Tebu International, Le Perray-en-Yvelines, France). Alternatively, C_6_-ceramide was from Matreya (Pleasant Gap, PA). N-butyldeoxynojirimycin (DNJ) was from Toronto Research Chemicals (North York, Canada). RPMI 1640 medium, antibiotics and fetal calf serum were purchased from Invitrogen (Cergy-Pontoise, France). All solvents of analytical grade were from Merck (Darmstadt, Germany) and other reagents were obtained from Sigma (L'Isle d'Abeau, France). Radiolabelled [choline-methyl-^14^C]-sphingomyelin was from New England Nuclear (Paris, France) and [ceramide-^3^H]-sphingomyelin was from CEA (Gif-sur-Yvette, France).

### Cell culture

Epstein-Barr virus (EBV)-transformed B lymphocytes from control individuals and from patients affected with NPD Type A (cell lines Tre and Elg) have previously been described and characterized with respect to sphingomyelin metabolism [15]. The transformed B cells derived from a NPD Type B patient (line MS1418), who is homozygous for a G to A nucleotide substitution at position 1799 leading to p.R600H in the aSMase protein, and the corresponding cell line transduced with an empty vector (cell line MS1418−) as well as the aSMase-transduced (cell line MS1418+) MS1418 cells have previously been reported [14], [15]. These cell lines were kindly provided by Dr. W. van Blitterswijk (Amsterdam, The Netherlands). The RLS lymphoid cell line, which is derived from a patient deficient for GM3 synthase activity [18] (OMIM 609056) was a kind gift from Dr. H. Wang (Middlefield, OH). Lymphoid cells were routinely grown in a humidified 5% CO_2_ atmosphere at 37°C in RPMI 1640 medium containing Glutamax (2 mmol/l), penicillin (100 U/ml), streptomycin (100 µg/ml) and 10% heat-inactivated fetal calf serum (FCS). Possible mycoplasma contaminations were monitored regularly by PCR.

### Flow cytometry analyses

Fas/CD95 cell surface expression was determined after incubation of cells for 30 min at 4°C with or without anti-CD95-phycoerythrin or an irrelevant antibody coupled to phycoerythrin (Beckman-Coulter). To allow study of phosphatidylserine externalisation, cells were labeled with Annexin V-fluorescein isothiocyanate (250 ng/ml) and propidium iodide (12.5 µg/ml) (Abcys, Paris, France) for 10 min at 4°C. The level of GM3 at the cell surface was examined on intact cells by using an anti-GM3 antibody from Seikagaku (Coger, Paris, France). Analyses were performed using a FACSCalibur flow cytometer (BD Biosciences).

### DNA sequencing

Total RNA was extracted from lymphoid cells and reverse-transcribed using Superscript II (Invitrogen). Part of the *ST3GAL5* (accession number NM_003896) cDNA encoding for GM3 synthase (E.C. number 2.4.99.9) was PCR-amplified using the following primer pair: 2GM3S, 5′-GGGCGCACCACTGTCTGACC-3′ and 2GM3AS, 5′-TCGGCCCCAGAACCTTGACTGA-3′, and Phusion High-Fidelity DNA polymerase (Ozyme, St-Quentin-en-Yvelines, France). Nucleotide sequencing was performed by MilleGen (Labège, France).

### Cell viability assay and morphological analyses

Viability was evaluated by the tetrazolium-based MTT assay. Cells were seeded in flat-bottomed 96-well plates for 24 or 48 hours at 37°C. 3-(4,5-Dimethylthiazol-2yl)-2,5-diphenyltetrazolium bromide (MTT, 0.5 mg/ml; Euromedex, Mundolsheim, France) was added during 3 hours. Formazan crystals were solubilized overnight at 37°C by adding 100 µl of solubilization buffer (HCl 0.01N, 10% SDS) and spectrophotometrically quantified using a microplate reader (wavelength = 590 nm).

Apoptosis was assessed by Hoechst staining as described previously [19]. The percentage of apoptotic cells was calculated as the number of apoptotic cells per one hundred total cells counted.

### Fluorogenic DEVD cleavage enzyme assay

After treatment, cell pellets were homogenized in an aqueous buffer made of 10 mM HEPES (pH 7.4), 42 mM KCl, 5 mM MgCl_2_, 0.5% CHAPS, 1 mM dithiothreitol, 1 mM PMSF, and 2 µg/ml leupeptin. Reaction mixtures contained 100 µl of cell lysates and 100 µl of 40 µM Ac-Asp-Glu-Val-Asp-7-amino-4-methylcoumarin (Ac-DEVD-AMC; Bachem, Voisins-le-Bretonneux, France) [19]. After 30 min incubation at room temperature, the amount of the released fluorescent product aminomethylcoumarin was determined using a Jobin-Yvon spectrofluorometer (at 351 and 430 nm for the excitation and emission wavelengths, respectively).

### Western blot analysis for caspase-3 processing

Analysis of caspase-3 cleavage was carried out by Western blot using the cell lysates prepared for DEVD cleavage enzyme assay as previously described [19]. Rabbit anti-caspase-3 was a gift from Dr. D. Nicholson (Merck-Frosst, Pointe-Claire, Quebec) or was provided by Cell Signaling (Ozyme). Proteins were visualized by ECL using anti-rabbit horseradish peroxidase-conjugated IgG (Bio-Rad, Marnes-la-Coquette, France).

### Quantitation of acid sphingomyelinase activity

Acid sphingomyelinase activity was determined as previously described [20]–[22] using [choline-methyl-^14^C]sphingomyelin (120,000 dpm/assay; specific activity 54 mCi/mmol) as substrate.

### Incubation of intact cells with radiolabelled sphingomyelin

Cells were incubated at 37°C for 24 h with medium containing 10% fetal calf serum and [ceramide-^3^H]sphingomyelin (10^6^ dpm/ml; specific activity 400 mCi/mmol) which was added as an ethanolic solution [22]. The cells were then washed and the lipids were extracted and resolved by analytical thin-layer chromatography on plates migrated in chloroform/methanol/water (100∶42∶6, by vol.). The distribution of the radioactivity on the plates was analyzed using a Berthold LB2832 radiochromatoscan. Unlabeled or radioactive lipid standards were used to identify the various metabolic products.

### Lipid analysis

As the incubation times with apoptogenic agents were short, the treatment with such agents was carried out in phosphate-buffered saline pH 7.4 (PBS). In typical experiments, 5.10^7^ cells were washed with buffer by centrifugation at 1000 rpm in round-bottomed 30 ml centrifuge tubes, taken up in 1 ml of buffer and kept at 37°C for 15 min prior to the addition of anti-Fas or C_6_-ceramide. The cellular metabolism was stopped by the addition of 20 ml of chloroform-methanol 1∶1 (v/v) and the lipids were extracted by stirring 3 h at 37°C. After centrifugation, the clear supernatants were transferred to flasks and evaporated to dryness at 40°C in a rotary evaporator (Büchi, Zurich, Switzerland). The lipids were dissolved in chloroform/methanol (1∶1), filtered to remove any non-soluble material, and partitioned three times in chloroform/methanol/PBS (1∶1∶0.7, by volume) as previously reported [23]. The gangliosides were recovered from the pooled upper phases by reverse-phase chromatography on styrene-divinylbenzene copolymer columns (Envi-Chrom™, Supelco, L'Isle d'Abeau, France) as described by Popa et al. [24]. The gangliosides were quantitated by the periodate-resorcinol method [25] and migrated on high performance thin-layer chromatography (HPTLC) plates in chloroform/methanol/0.2% CaCl_2_ (60∶35∶8, by volume) along with known standards. Gangliosides species were identified by immunostaining on thin-layer plates as described [26] using the following monoclonal antibodies (Mabs) : 4E6 specific for GD3, GMR28 directed to GM2 (gift from Dr. Tadashi Tai, Tokyo Metropolitan Institute of Medical Science, Tokyo, Japan) and E1 reacting with GM1 (kindly provided by Dr. M. Alfonso, Center of Molecular Immunology, Havana, Cuba). The lower phases of partition were evaporated to dryness under nitrogen, taken up in chloroform and the lipid classes were separated by column chromatography on aminopropyl silica gel cartridges (LC-NH_2_, Supelco) according to Bodennec et al. [27].

### Statistical analyses

Data are presented as means ± S.E.M. The Student's *t*-test was used for statistical analysis (*, p<0.05; **, p<0.01).

## Results

### Fas cross-linking and exogenous ceramide treatment trigger different ganglioside production in normal as well as Niemann-Pick disease lymphoid cells

We have investigated the generation of gangliosides in EBV-transformed lymphoblasts derived from control subjects and patients affected with NPD type A (the most severe form of NPD) [15]. Among these cell lines, we have used the MS1418 cell line deficient in aSMase activity [28], as well as the MS1418+ cell line that re-expresses aSMase after retrovirus-mediated gene transfer (Table 1) [14].

**Table 1:** *In vitro* aSMase activity and lysosomal sphingomyelin turnover in cultured lymphoblasts.

Cell line	aSMase activity	Undegraded sphingomyelin
	(nmol per hr/mg of protein)	(% of total)
Control	3.22±0.23	25
Tre	0.02±0.01	95
MS1418	0.10±0.01	90
Corrected MS1418+	2.30±0.20	35

Acid sphingomyelinase activity was determined on cell lysates using [choline-methyl-^14^C]sphingomyelin. Undegraded sphingomyelin was measured after incubation of intact cells with [ceramide-^3^H]sphingomyelin (see [22]).

**Figure 1:**
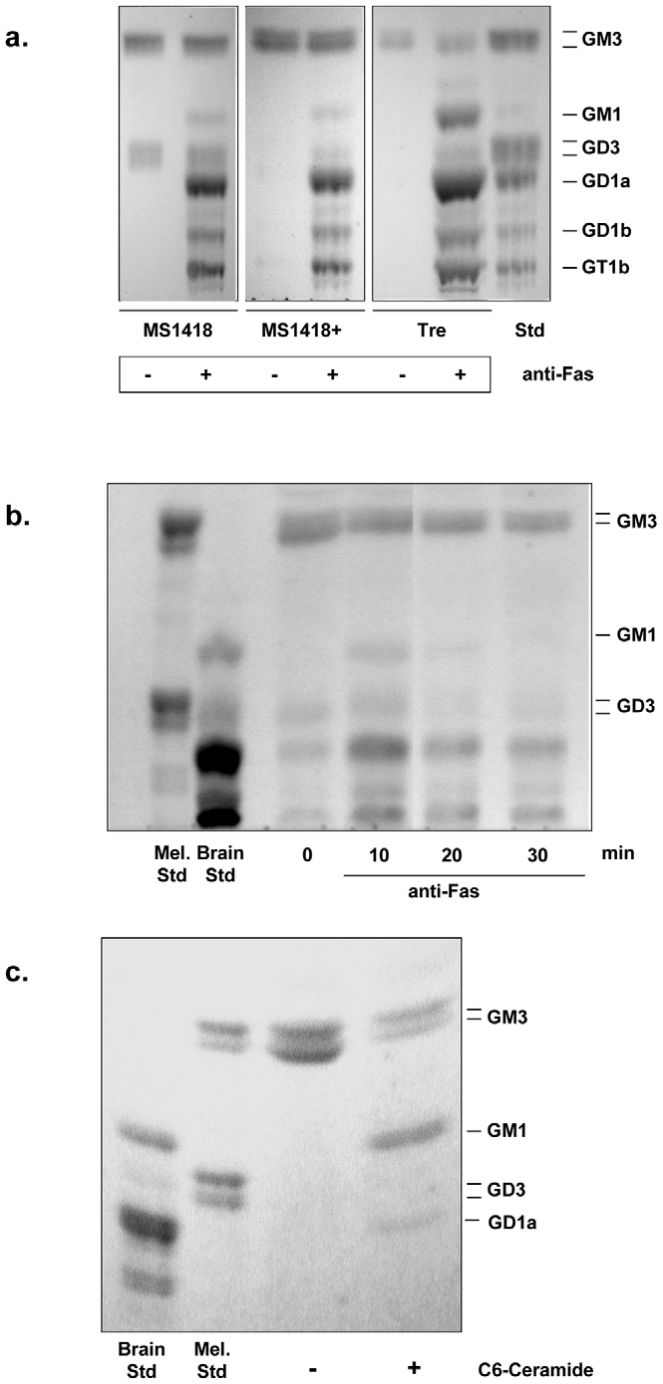
Production of gangliosides in Niemann-Pick disease lymphoid cells upon treatment with anti-Fas or C_6_-ceramide. (*a*) Ganglioside production in Niemann-Pick lymphoblasts after treatment with anti-Fas antibody (200 ng/ml) for 15 min. Gangliosides were analyzed by HPTLC. The standard (Std) represents a mixture of bovine brain and melanoma gangliosides. For each cell line, the same number (50×10^6^) of untreated and anti-Fas-treated cells was used. Lipids were extracted and loaded on the plate. (*b*) MS1418 cells (25×10^6^) were incubated at 37°C for the indicated times with anti-Fas (500 ng/ml). Then, gangliosides were extracted and analyzed by HPTLC. Std represents standard gangliosides from melanoma (Mel.) or bovine brain. (*c*) Tre NPD lymphoblasts (25×10^6^) cells were treated for 10 min at 37°C with 50 µM of C_6_-ceramide dissolved in ethanol. Then, gangliosides were purified and analyzed by HPTLC.

**Figure 2:**
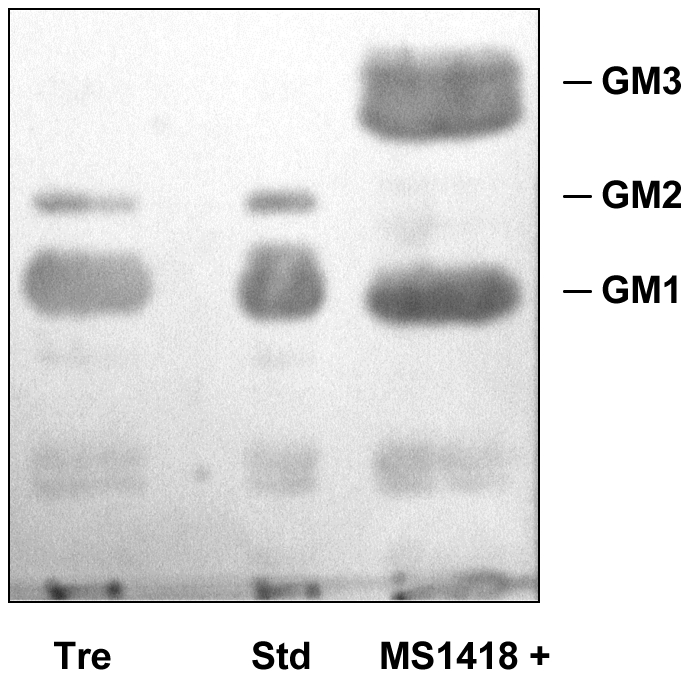
Thin-layer chromatography immunostaining with a mixture of anti-GM3, anti-GM2 and anti-GM1 Mabs of gangliosides of Tre and MS1418+ cell lines after 10 min treatment with anti-Fas. Std represents standard gangliosides from bovine brain. The amounts of gangliosides applied on the HPTLC plate were one tenth of those visualized by resorcinol-HCl in Fig. 1*a*.

The production of gangliosides was analyzed after Fas ligation by thin-layer chromatography. As illustrated in Fig. 1*a*, Niemann-Pick lymphoblasts (MS1418 and Tre cell lines) synthesized within 5 to 15 min several new species of gangliosides of the A-series (GM2, GM1, GD1a) with a whole ganglioside pattern reminiscent of that in brain [29]. Identification of gangliosides was carried out by immunostaining on thin-layer plates [26] after migration of the gangliosides, using a mixture of monoclonal antibodies known to react respectively with GM3, GM2 and GM1 as shown in Fig. 2. Identification of the other species, done by co-migration with authentic standards, indicated the presence of GD1a, GD1b, and GT1b, GD3 not being anymore the predominant ganglioside. An intriguing feature was the migration of these additional gangliosides as single bands (see also the lane of standards in Fig. 1*a*) instead of the doublets that can be seen with GM3 and GD3 as in untreated cells. Single bands of gangliosides from normal human tissues are usually seen only with brain and red cells. It should be noted that GM3 level was not affected by anti-Fas treatment, suggesting that the generation of complex gangliosides did not originate from the pre-existing GM3 pool (Fig. 1*a*).

**Figure 3:**
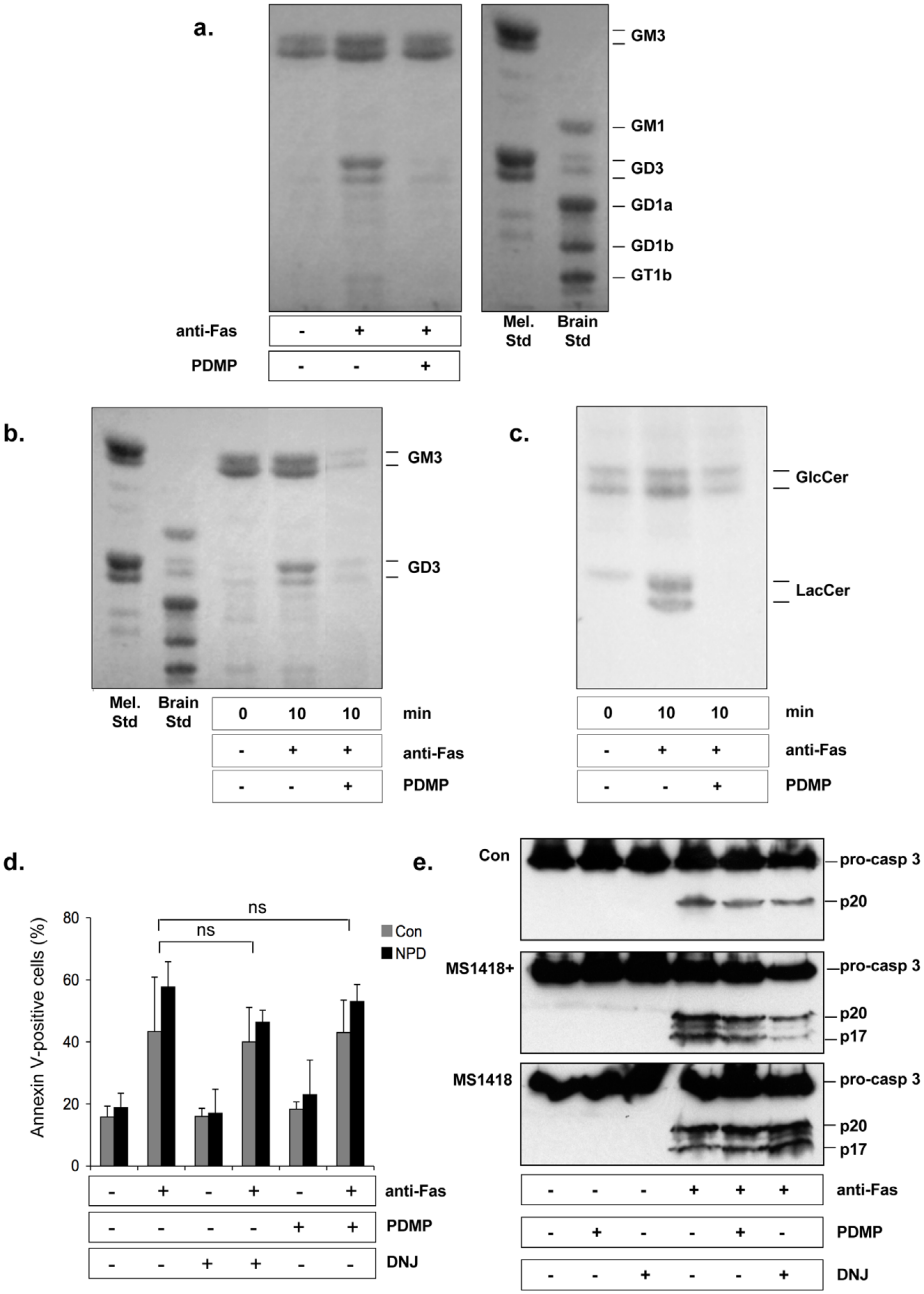
Pharmacological inhibition of ganglioside production does not prevent from anti-Fas-induced apoptosis. (*a*) Production of gangliosides in control lymphoblasts after treatment with 200 ng/ml anti-Fas antibody for 10 min with or without pre-treatment with 10 µM PDMP. Melanoma (Mel. Std) and bovine brain (Brain Std) ganglioside mixtures were used as standards. (*b*) Effect of PDMP on the production of gangliosides by NPD (MS1418) cells that were incubated as described in (*a*). (*c*) Effect of PDMP on the production of neutral glycosphingolipids by NPD (MS1418) cells that were incubated as described in (*a*). GlcCer, glucosylceramide; LacCer, lactosylceramide. (*d*) Control (Con) and NPD lymphoblasts were pre-incubated for 24 h with or without 10 µM PDMP or 400 µM DNJ, and treated without or with 100 ng/ml anti-Fas antibody for 5 h under serum-free conditions. Cell surface exposure of phosphatidylserine was determined by flow cytometry using annexin V-FITC/PI labelling (ns, not significant). (*e*) Control (Con), NPD (MS1418) and aSMase-transduced NPD (MS1418+) cells were incubated as indicated in (*d*) and cell extracts were subjected to 15% SDS-PAGE and immunoblotted with anti-caspase-3 antibody. Migrations indicated are: pro-caspase-3, full-length inactive caspase-3; p20 and p17, active subunits.

Interestingly, the aSMase-transduced NPD (line MS1418+) ganglioside pattern after anti-Fas treatment was comparable to that of the untransduced MS1418 NPD cell line (Fig. 1*a*). The ganglioside generation was quantitatively very important, up to 50-fold above the control, but it was transient as most of the neosynthesized gangliosides disappeared after 30 min and the ganglioside pattern was back to normal within 60 min beyond treatment (Fig. 1*b*). In addition, ganglioside production was also observed when cells were treated with exogenous C_6_-ceramide (Fig. 1*c*). An interesting observation is that, after anti-Fas or cell-permeable ceramide treatment, EBV-transformed lymphoblasts derived from control subjects showed a rapid synthesis of gangliosides, but the increase involved mostly the GM3 and GD3 ganglioside species already found in the control cells, in striking contrast with the ganglioside profile seen with NPD cells (see Fig. 3*a*). This different response in the ganglioside species neosynthesized between control and aSMase-deficient (but also aSMase-transduced) cell lines is not yet understood. It is noteworthy to point out that increased levels of the lactosylceramide precursor were seen concomitantly with the rapid increase in ganglioside biosynthesis (see Fig. 3*c*).

Notwithstanding, our data markedly differ from those previously published [6], [9] as i) Niemann-Pick lymphoblasts are fully capable of producing GD3, and ii) GD3 is far from being the unique ganglioside produced during Fas-induced apoptosis.

### Fas cross-linking and exogenous ceramide treatment induce apoptosis, and caspase-3 activation analogously in normal and Niemann-Pick disease lymphoid cells

**Figure 4:**
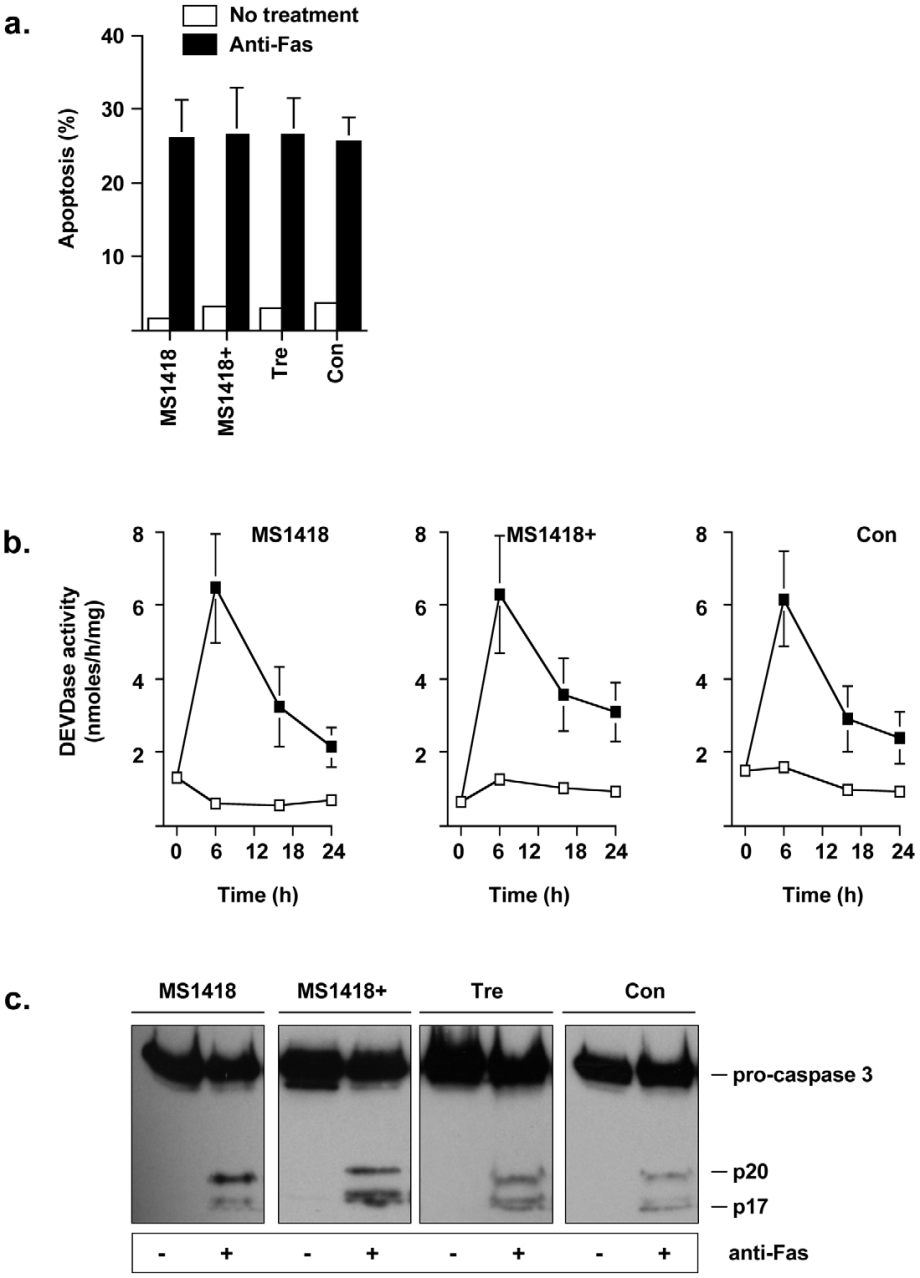
Control and Niemann-Pick disease lymphoblasts are equally sensitive to anti-Fas-induced apoptosis. (*a*) Control, NPD (MS1418 and Tre cell lines), and aSMase-transduced NPD (MS1418+ cell line) lymphoblasts were incubated in serum-free conditions with or without 100 ng/ml anti-Fas for 5 h. The percentage of apoptotic cells was evaluated by the DNA-specific fluorochrome Hoechst 33258. Nuclei were visualized by fluorescence microscopy, and a minimum of 300 cells were scored. Mean values and standard deviations for each sample from at least three different experiments are shown. (*b*) DEVDase activity was measured with the fluorogenic substrate Ac-DEVD-AMC in extracts from NPD (MS1418 and Tre cell lines), and the aSMase-transduced NPD (MS1418+ cell line) lymphoblasts treated for the indicated times without (open squares) or with 100 ng/ml anti-Fas antibody (filled squares). Results are means ± S.D. of at least three independent experiments. (*c*) Extracts from control, NPD (MS1418 and Tre cell lines), and aSMase-transduced NPD (MS1418+ cell line) lymphoblasts treated with anti-Fas antibody for 5 h were subjected to 15% SDS-PAGE and immunoblotted with anti-caspase-3 antibody. Migrations indicated are: pro-caspase-3, full-length inactive caspase-3; p20 and p17, active subunits.

We next compared the sensitivity to apoptosis of the NPD lymphoid cells. As we have previously reported [15], treatment with anti-Fas antibody induced apoptosis equally well in control, NPD (MS1418 and Tre cell lines), and corrected NPD MS1418+ cell line (Fig. 4*a*). We also examined whether aSMase influenced processing of executioner caspases (such as caspase-3 and -7), the proteases that drive the effector phase of apoptosis, and which are activated during Fas-induced apoptosis. To monitor executioner caspase activity, we utilized the fluorogenic substrate, Ac-DEVD-AMC, which corresponds to the cleavage site found in various caspase-3 and -7 targets. In control, NPD MS1418 and corrected NPD MS1418+ cell lines, we observed similar time-dependent increases in DEVDase activity (Fig. 4*b*) that correlated with the onset of apoptosis. Proteolytic cleavage of caspase-3 was also examined by Western blotting, and Fig. 4*c* illustrates the processing of caspase-3 into its respective active forms regardless of the aSMase status of the cell lines used. These findings clearly indicate that normal and NPD lymphoblast cells are equally sensitive to Fas-induced apoptosis. Similar results were found when cells were treated with exogenous C_6_-ceramide (data not shown).

### Blocking ganglioside production does not abrogate apoptosis induced by Fas ligation in both normal and Niemann-Pick disease lymphoid cells

Thereafter, in order to establish the relevance of ganglioside production during Fas-induced apoptosis in NPD lymphoblast cells, we decided to study the effect on apoptosis of two specific inhibitors of glucosylceramide synthase, PDMP and DNJ [30], [31]. As shown in Table 2, incubation of the Tre NPD cell line with DNJ and PDMP raised the intracellular concentration of ceramide to the detriment of endogenous glucosylceramide. Similar results were found with the other cell lines tested regardless of the aSMase status.

**Table 2:** Neutral sphingolipid percent distribution in NPD Tre cells pre-incubated with the indicated concentrations of NDJ or PDMP.

Neutral sphingolipid (% of total)		
	Glucosylceramide	Ceramide
Untreated	54	41
DNJ (100 µM)	19	78
DNJ (200 µM)	14	83
DNJ (400 µM)	10	88
PDMP (10 µM)	8	90
PDMP (20 µM)	7	90
PDMP (40 µM)	7	92

Lipids were separated by HPTLC and their concentrations were determined after acid hydrolysis using fluorescamine as previously described [37].

As expected, the ganglioside profile was markedly altered when cells were pretreated with PDMP. As illustrated in Fig. 3*a*, there was indeed a strong reduction in ganglioside generation upon Fas ligation in control lymphoblasts pre-incubated with 10 µM PDMP. NPD and reconstituted NPD lymphoblasts (MS1418 and MS1418+ cell lines, respectively) exhibited a similar profile (Fig. 3*b*). Comparable findings were also observed when glucosylceramide synthase was inhibited by DNJ (data not shown). Both the increase of the ganglioside precursor lactosylceramide (Fig. 3*c*) and GD3, following anti-Fas treatment, were completely abolished by PDMP.

In addition, pre-treatment with PDMP or DNJ did not impede Fas-induced apoptosis as evidenced by the assessment of phosphatidylserine translocation (Fig. 3*d*) as well as by the processing of executioner caspase-3 which underwent efficiently despite the inhibition of glycosphingolipid synthesis (Fig. 3*e*). These results clearly show that inhibition of glucosylceramide synthase does not confer resistance to Fas-mediated apoptosis in lymphoblasts, and strongly suggest that gangliosides are unlikely to play a role in transducing this apoptotic signal.

**Figure 5:**
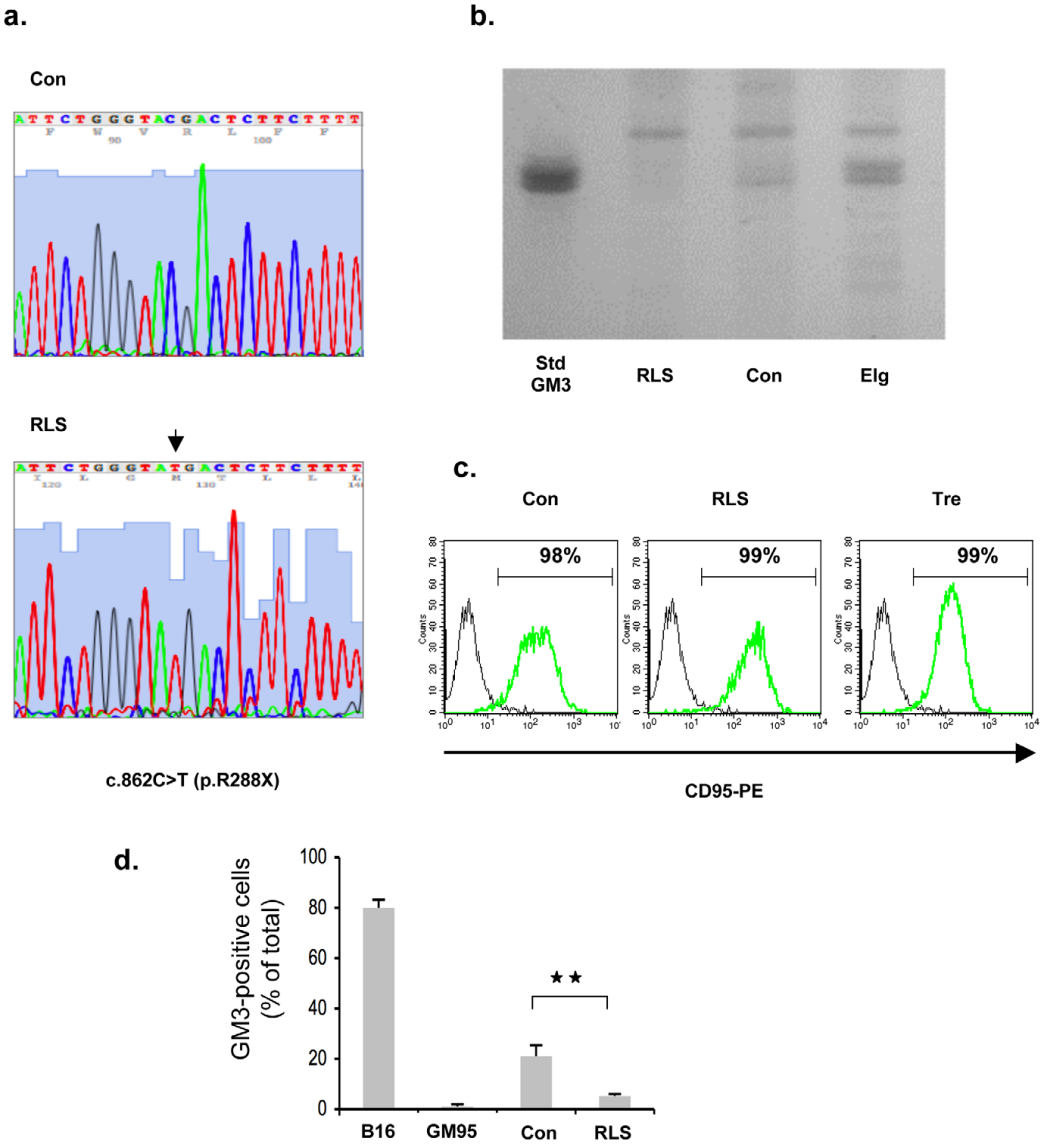
Characterization of the GM3 synthase-deficient lymphoid cell line. (*a*) Sequencing of the *ST3GAL5* cDNA prepared from control (Con) and the RLS (GM3 synthase-deficient) cell lines. The arrow indicates the c.862C>T point mutation. (*b*) HPTLC analysis of gangliosides isolated from control (Con), the RLS and Elg (NPD type A) cell lines. GM3 was used as a standard lipid. (*c*) Cell surface expression of Fas/CD95. Intact control (Con), RLS and Tre (NPD type A) cell lines were incubated with an anti-CD95 (green line) or an irrelevant (black line) antibody. Labelling was evaluated by flow cytometry. (*d*) Cell surface expression of GM3 in control (Con) and RLS lymphoblasts, as evaluated by flow cytometry. Control murine melanoma (B16 cell line) and glucosylceramide synthase-deficient murine melanoma (GM95) cells were used as controls.

Finally, the susceptibility of GM3 synthase-deficient cells to Fas-induced cell death was investigated. For this purpose, lymphoid cells from a patient with a genetic deficiency of ganglioside GM3 synthase activity [18] were used. Analysis of the cDNA encoding ST3 beta-galactoside alpha-2,3-sialyltransferase 5 gene (*ST3GAL5* or *SIAT9*) showed homozygosity for the c.862C>T nonsense mutation which produces a premature termination codon at position R288 (Fig. 5a). This genetic defect was accompanied by the absence of detectable GM3 as evaluated by thin-layer chromatography of total cellular lipids (Fig. 5*b*); similar results were obtained by measuring the expression of GM3 at the cell surface using flow cytometry (Fig. 5*d*). Of note, the cell surface expression of the Fas/CD95 receptor remained unaltered (Fig. 5*c*).

**Figure 6:**
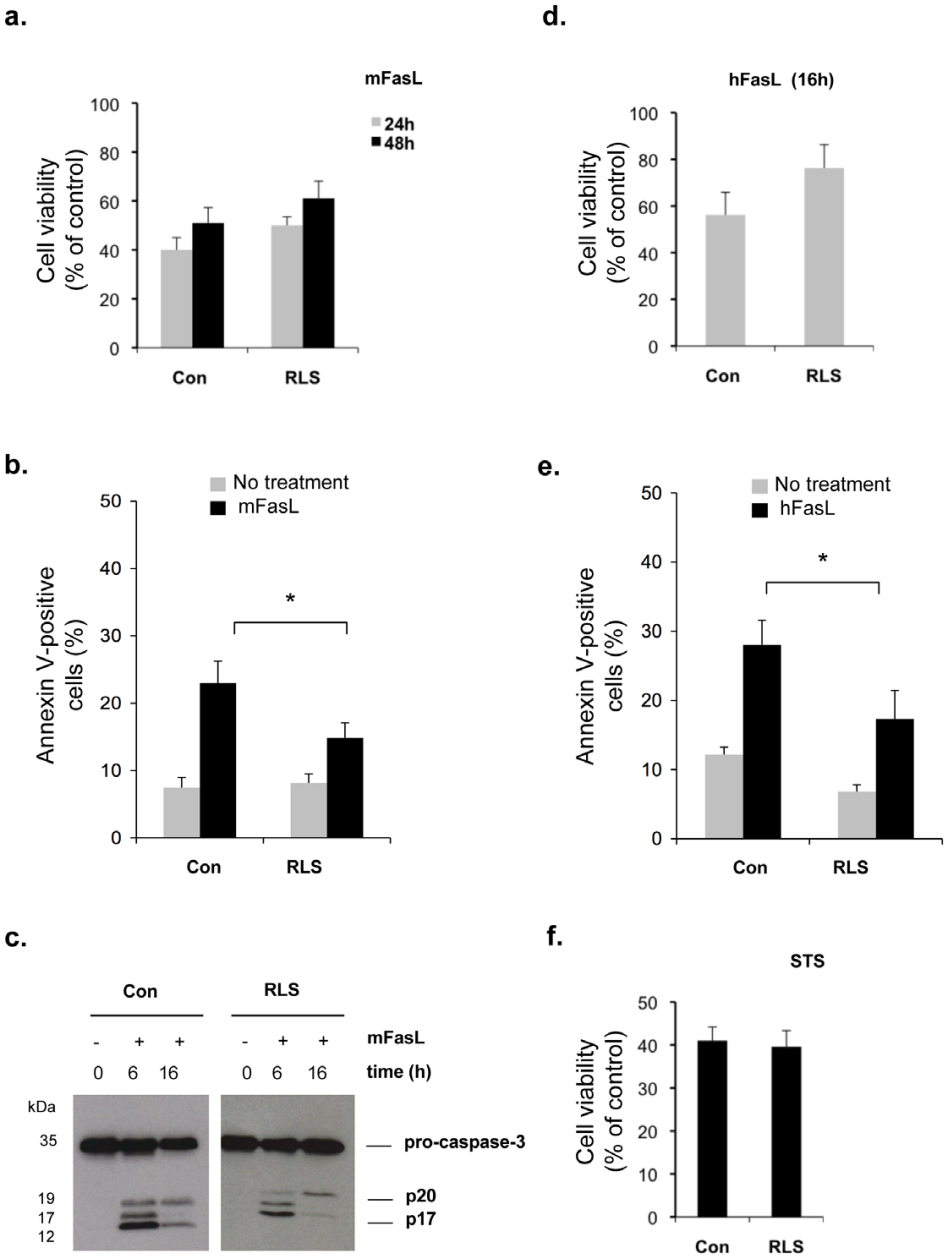
Susceptibility of GM3 synthase-deficient lymphoid cells to apoptosis inducers. (*a–c*), Control (Con; 3 different cell lines) and RLS (GM3 synthase-deficient) cells were treated with 1 µg/ml mFasL. After 24 or 48 h incubation, cell viability was assessed by the MTT test (*a*) or cell surface exposure of phosphatidylserine was determined by flow cytometry after a 24 h incubation (*b*). Data are means ± SEM of at least 3 determinations. Alternatively, processing of caspase-3 was monitored by Western blot after the indicated time of incubation (*c*). (*d*) and (*e*), Control and RLS cells were incubated for 16 h with 1 µg/ml hFasL. Then, cell viability (*d*) and cell surface exposure of phosphatidylserine (*e*) were determined. Data are means ± SEM of at least 3 determinations. Note that, for (*b*) and (*e*), comparable results were obtained when analyzing the annexin V-positive PI-negative cell population. (*f*), Cell viability of lymphoid cells after treatment for 24 h with 100 nM of staurosporine (STS).

When these and control lymphoblasts were challenged with either murine or human FasL, no significant difference in loss of cell viability was seen (Figs. 6*a* and 6*d*). As a control condition, incubation of mutant and control cells with staurosporine, a protein kinase inhibitor triggering the mitochondrial pathway of apoptosis, resulted in similar cytotoxic effects (Fig. 6*f*). However, phosphatidylserine translocation evaluated as a feature of apoptosis was less prominent in the GM3 synthase-deficient lymphoblasts than in control cells (Figs. 6*b* and 6*e*). Nevertheless, caspase-3 processing was clearly activated in the mutant cell line as evidenced by the appearance of cleaved forms (Fig. 6*c*).

## Discussion

Fas-induced activation of an aSMase leading to generation of GD3, a glycolipid (possibly also mitochondrial) raft constituent, has been associated with apoptosis of lymphoid cells [6], [9], [32]. It has been suggested that activation of this aSMase would induce the accumulation of GD3, which in turn would mediate apoptosis by targeting mitochondria [6], [8], [9]. Nevertheless, aSMase involvement in Fas-induced apoptosis is still controversial. Whereas some authors reported that NPD lymphoblasts are resistant to Fas-mediated cell death [9], others have found no differences [14], [15]. In addition, thymic cells from *Smpd1−/−* mice display no resistance to apoptosis *in vivo* or *ex vivo* supporting the notion that aSMase is not necessary to mediate apoptosis in lymphoid cells [16] [11], [12], [28]. Our studies confirm previous results [14], [15] that Fas cross-linking trigger similar apoptotic hallmarks in normal and aSMase-deficient cell lines.

The present findings show that, at least in lymphoid cells, Fas can indeed trigger the production of gangliosides but this production is not requisite for induction of cell death. Such a generation of gangliosides appeared i) to be an early event (occurring within the minutes following Fas ligation), ii) to involve several gangliosides, not only GD3, iii) to rely on the ceramide to glucosylceramide conversion, and iv) to involve the entire pathway of glycosylation since the levels of the ganglioside precursor lactosylceramide also increased along with gangliosides. The present observations also demonstrate that aSMase activation is not accountable for ganglioside generation in cultured lymphoblasts as originally described [6], [9].

**Figure 7:**
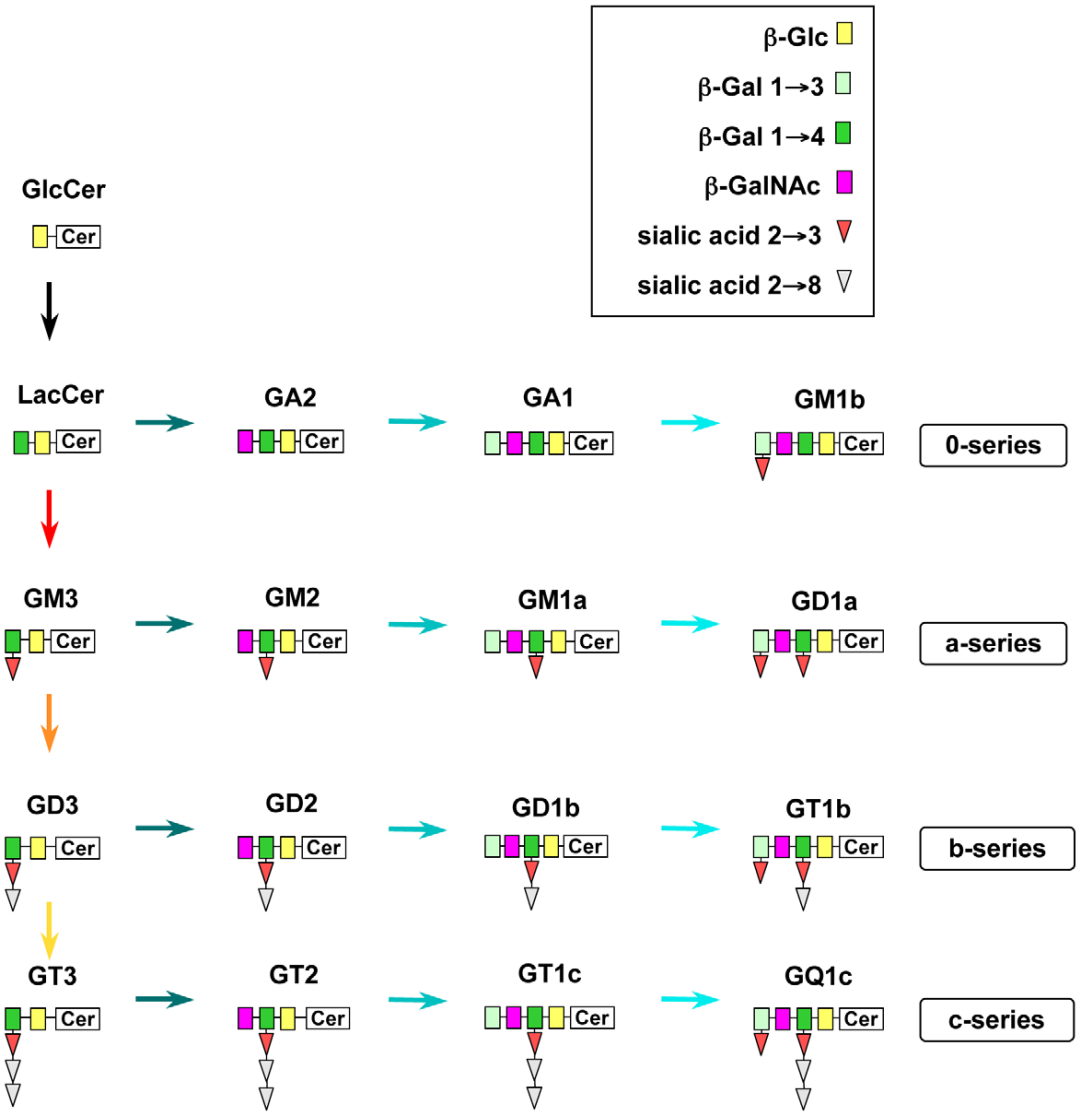
Schematic view of ganglioside biosynthesis in mammalian cells. Sequential addition of sialic acid residues to lactosylceramide (LacCer) by the specific sialyl-transferases I, II and III produces the monosialo, disialo and trisialo-gangliosides GM3, GD3 and GT3, respectively. These glycolipids serve as precursors for the a, b and c series of gangliosides, which are formed by stepwise glycosylation, i.e., by addition of N-acetyl-beta-galactosamine (GalNAc), beta-galactose (Gal) and sialic acid by a GalNAc-transferase, a galactosyl-transferase and a sialyl-transferase, respectively.

Of importance is the observation that GD3 was not the only ganglioside produced upon Fas ligation. Conversely, we found within a few minutes an accumulation of b-series (e.g., GD1b and GT1b) as well as a-series (e.g., GM1a and GD1a) gangliosides (see Figure 7 and the accompanying legend for the biosynthetic pathways of gangliosides), demonstrating that GalNAc-transferase and other transferases are present, but normally inactive in these cells. It should be pointed out that the relevant hydrolases also should be present in cells but in the inactive form since all *de novo* synthesized gangliosides totally disappeared in less than one hour. No traces of gangliosides was found in the buffer, thus ruling out any shedding to explain this rapid disappearance.

Disruption of the *GalNAcT (B4galnt1)* gene [33] has been shown to block the synthesis of complex gangliosides and results in the expression of the simple gangliosides GM3 and GD3. Despite the enormous amounts of GM3 and GD3 accumulated, these mutants are viable, with a normal life span and a central nervous system that is largely intact both morphologically and functionally [34], [35]. Furthermore, GD3 synthase-deficient (*St8sia1* knockout) mice that are devoid of the b-series structures, including GD3, GD2, GD1b, GT1b and GQ1b, mainly accumulate a-series gangliosides such as GM2, GM1 and GD1a [29], [36]. These b-series ganglioside-lacking mice are viable, have normal growth, and do not exhibit gross behavioral abnormalities [29], [36]. More importantly, thymocytes from the wild-type and the GD3 synthase-deficient mice were used for the induction of apoptosis by anti-Fas antibody, but no reduction in the sensitivity to Fas-induced apoptosis could be detected [29]. Hence, deletion of the b-series gangliosides including GD3 does not seem to alter Fas-induced apoptosis in thymocytes clearly arguing against a role for GD3 in mediating Fas-induced apoptosis. In agreement, we have found that blockade of ganglioside production by inhibiting glucosylceramide synthase by PDMP or DNJ does not impede apoptosis.

Furthermore, cells that are genetically unable to synthesize GM3 (and more complex gangliosides) underwent Fas-induced effector caspase activation and cell death. Nonetheless, because these mutant cells exhibited less phosphatidylserine exposure than the control cell lines, the possibility that (GM3) gangliosides are somehow involved as key plasma membrane constituents in this phenomenon and the overall response to Fas ligation cannot be ruled out. In addition, the possibility that such a behavior is restricted to this particular mutant cell line (but not present in other GM3 synthase-deficient cell lines) cannot be excluded as some interindividual variations between control cell lines in the degree of sensitivity to Fas-induced toxicity were observed (data not shown).

In summary, the present investigation suggests that aSMase is not required for production of GD3 and that gangliosides do not play an essential role in transducing Fas-induced apoptosis of cultured lymphoblasts. The data presented here clearly question the current model for the transduction of apoptosis by which the ganglioside GD3 would mediate Fas-induced apoptosis in lymphoblasts. An intriguing finding of our study is the capacity of NPD-derived lymphoid cells to synthesize during apoptosis several ganglioside species that are usually not seen during the normal life span of those cells.
